# Heart Rate Variability and Exceptional Longevity

**DOI:** 10.3389/fphys.2020.566399

**Published:** 2020-09-17

**Authors:** Adrián Hernández-Vicente, David Hernando, Alejandro Santos-Lozano, Gabriel Rodríguez-Romo, Germán Vicente-Rodríguez, Esther Pueyo, Raquel Bailón, Nuria Garatachea

**Affiliations:** ^1^GENUD (Growth, Exercise, NUtrition and Development) Research Group, University of Zaragoza, Zaragoza, Spain; ^2^Department of Physiatry and Nursing, Faculty of Health and Sport Sciences (FCSD), University of Zaragoza, Huesca, Spain; ^3^BSICoS Group, Aragón Institute for Engineering Research (I3A), IIS Aragón, University of Zaragoza, Zaragoza, Spain; ^4^CIBER de Bioingeniería, Biomateriales y Nanomedicina (CIBER-BBN), Zaragoza, Spain; ^5^i+HeALTH, European University Miguel de Cervantes, Valladolid, Spain; ^6^Research Institute of Hospital 12 de Octubre (“i+12”), Madrid, Spain; ^7^Faculty of Physical Activity and Sports Sciences, INEF, Universidad Politécnica de Madrid, Madrid, Spain; ^8^CIBERFES, Madrid, Spain; ^9^Centro de Investigación Biomédica en Red de Fisiopatología de la Obesidad y Nutrición (CIBER-Obn), Madrid, Spain; ^10^Instituto Agroalimentario de Aragón -IA2- (CITA-Universidad de Zaragoza), Zaragoza, Spain

**Keywords:** electrocardiography, autonomic nervous system, parasympathetic nervous system, heart rate, heart rate variability, mortality, centenarians, aging

## Abstract

Centenarians are the paradigm of human extreme longevity and healthy aging, because they have postponed, if not avoided, mayor age-related diseases. The purpose of this study was to investigate potential differences in resting heart rate variability (HRV) between young adults, octogenarians, and centenarians and assess whether HRV variables are predictors of all-cause mortality in centenarians. To this end, three groups of participants: young adults (*N* = 20; 20.6 ± 2.3 years), octogenarians (*N* = 18; 84.1 ± 2.6 years), and centenarians (*N* = 17; 101.9 ± 1.9 years) were monitored for 15 min at rest (seated, without moving or talking) to measure RR intervals, from which HRV was evaluated. Our results showed a clear decrease with age in the main parasympathetic HRV variables, as well as in the standard deviation (SD) of the RR series [SD of normal-to-normal interval (SDNN)] and in low frequency (LF) heart rate (HR) oscillations, although differences between octogenarians and centenarians did not reach statistical significance. In 14 centenarians followed until death, only SDNN showed significant correlation (*ρ* = 0.536; *p* = 0.048) with survival prognosis. Additionally, SDNN <19 ms was associated with early mortality (≤1 year) in centenarians (Hazard Ratio = 5.72). In conclusion, HRV indices reflecting parasympathetic outflow as well as SDNN and LF all present an age-related reduction, which could be representative of a natural exhaustion of allostatic systems related to age. Moreover, low SDNN values (<19 ms) could be associated with early mortality in centenarians. HRV seems to play a role in exceptional longevity, which could be accounted for by centenarians’ exposome.

## Introduction

Heart rate variability (HRV) is defined as “the oscillation in the interval between consecutive heart beats” (Malik et al., 1996). HRV is the result of the interaction of multiple regulatory mechanisms that operate at different time scales, including long-term mechanisms like circadian rhythms, core body temperature, or metabolism and short-term mechanisms involving the autonomic, cardiovascular, and respiratory systems (Shaffer and Venner, 2013). Short-term spectral analysis of HRV usually reveals at least two frequency components, a low frequency (LF) component (0.04–0.15 Hz) and a high frequency (HF) component (>0.15 Hz; Malik et al., 1996). These components have been widely used to measure sympathetic and parasympathetic nervous systems, although their underlying physiological mechanisms are still unclear and a matter of debate (Billman, 2013).

In the last decades, several studies have reported that HRV decreases with age, suggesting an age-dependent decline in autonomic nervous system (ANS) activity in geriatric patients (Craft and Schwartz, 1995; Piccirillo et al., 1995). The majority of these studies have been mainly performed in older adults up to 80–85 years old, whereas older adults over age 85 have not received much attention. Centenarians represent the survival tail of the population (with a lifespan at least 15–20 years longer than the average westerner) and a model of healthy aging (Christensen et al., 2008). Indeed, centenarians escaped the diseases of the pre-antibiotic era and have postponed/avoided aging-related diseases as well as their fatal consequences (Salvioli et al., 2008).

The study of centenarians constitutes a fascinating research into the characteristics that allow individuals to attain an exceptionally long lifespan. Few works have studied HRV in centenarians. Piccirillo et al. (1998) and Paolisso et al. (1999) found that centenarians present higher power in HF heart rate (HR) oscillations and lower power in LF than old adults (75–100 years old in Paolisso et al. and 81–100 years old in Piccirillo et al.), suggesting age-related increase in parasympathetic activity and reduction in sympathetic activity. These results are in line with those obtained by Zulfiqar (2010) who enrolled subjects up to 99 years old and demonstrated that parasympathetic time-domain HRV measures decrease with age, reaching a nadir in the 7th–8th decade. From the 8th decade, these HRV measures are shown to rise, with the authors proposing this reversal of the decrease in parasympathetic function as a key determinant of longevity. In contrast, another study conducted in centenarians linked HRV with mortality during 4-year follow-up, showing that among all frequency-domain variables only higher LF/HF ratio was associated with survival (Shimizu et al., 2002).

Eight out of 10 centenarians are women (Teixeira et al., 2017). In 2016, Koenig and Thayer (2016) published a meta-analysis with 63,612 participants (31,970 females), revealing that: although adult women showed greater mean HR (MHR) than adult male, the female heart is characterized by a dominance of vagal activity (greater HF) and lower standard deviation (SD) of normal-to-normal intervals (SDNN). However, these sex differences may disappear in older adults (Voss et al., 2015; Koenig and Thayer, 2016), as a consequence of a variety of age-related changes such as: endocrine, brain structure, brain perfusion, or behavioral differences.

Due to the lack of current evidence and the discrepancies in the reported outcomes, the present study aimed at investigating potential differences in women’s HRV between young adults, octogenarians, and centenarians and assess whether HRV variables can predict all-cause mortality in centenarians followed up until the time of death.

## Materials and Methods

### Participants

Women aged 18–26 years in the group of young adults, 80–90 years in the group of octogenarians, and ≥100 years in the case of centenarians were included in the study. Due to the low number of centenarians, four men were additionally included in this group. In total, the young adults, octogenarians, and centenarians groups contained 20, 18, and 17 subjects, respectively. Exclusion criteria included the following: subjects going through an acute disease, suffering from heart diseases (e.g., heart failure or atrial fibrillation), or being on cardiac medication. Subjects who had a stroke or were suffering from chronic diseases such as diabetes, hypertension, chronic obstructive pulmonary disease, osteoarthritis, dementia, Parkinson’s, or thyroid diseases were included in the study because of their high prevalence in the last decades of life. The study was approved by the Clinical Research Ethics Committee of the University Hospital of Alcorcón (ID of the approval: 16/50) and was conducted adhering to the Declaration of Helsinki. After a clear explanation of the potential risks of the study, all volunteers (or their legally responsible for older adults with cognitive problems) provided written informed consent to participate in the study.

### Experimental Design

All the subjects completed one test session. Prior to the test session, subjects were asked to adhere to the following instructions: (1) avoid exercise or strenuous physical activity the day before the test; (2) drink plenty of fluids over the 24 h period preceding the test; (3) get an adequate amount of sleep (6–8 h) the night before the test; (4) avoid substances such as tobacco, alcohol, or stimulants (caffeine, theine, taurine, etc.) in the 8 h before the test; (5) avoid food for 3 h prior to taking the test; and (6) wear comfortable, loose-fitting clothing. All the subjects were tested in an environmentally controlled room (22–23°C) between 9:00 and 13:00 h. They were monitored for 15 min at rest (seated, without any movement or talking) to measure RR intervals. RR intervals were recorded on a beat-to-beat basis by using an HR monitor (RS800, Polar Electro Oy, Kempele, Finland) with a sampling frequency of 1,000 Hz, thus providing an accuracy of 1 ms for each RR period. This device has been recently validated, showing to provide comparable performance with respect to the electrocardiogram when analyzing HRV at rest (de Rezende Barbosa et al., 2016; Hernando et al., 2016).

### HRV Variables

HRV variables have commonly used to assess sympathetic and parasympathetic nervous systems. The LF component of HRV is assumed to provide information on cardiac sympathetic and parasympathetic neural activity, together with other regulatory mechanisms and baroreflex (Eckberg, 1997). The HF component, on the other hand, is assumed to be vagally mediated and driven by respiration, measuring the so-called respiratory sinus arrhythmia (RSA; Berntson et al., 1993). Based on these assumptions, the ratio of LF to HF (LF/HF) has been proposed to quantify the relationship between sympathetic and parasympathetic activities (i.e., the sympatho-vagal balance; Malik et al., 1996).

However, although these spectral indices are well-standardized, their physiological interpretation has been criticized. This especially applies to the relationship between LF power and cardiac sympathetic regulation (Eckberg, 1997; Billman, 2013; Reyes del Paso et al., 2013), with LF power decreasing during situations expected to increase sympathetic activity, such as exercise or myocardial ischemia, and lack of correlation between direct recording of sympathetic nerve activity and LF power in either healthy subjects or patients with heart failure. The interpretation of HF power has been also challenged, especially when the respiratory rate does not fall within the HF band (0.15–0.4 Hz; Laborde et al., 2017). Different approaches have been proposed to overcome this limitation by redefining the HF band (Bailón et al., 2007; Varon et al., 2018). It has also been suggested that sympathetic neural activity may modulate the HF component (Billman, 2013). Therefore, the physiological interpretation of the LF/HF ratio is unclear and likely underestimates the complex interactions between the sympathetic and parasympathetic regulation of HR (Billman, 2013).

Normalized LF power (LFn) represents the proportional contribution of sympathetic modulation (Malik et al., 1996), in the same way and with the same limitations as LF/HF ratio represents sympatho-vagal balance. A mathematical relationship exists between LFn and LF/HF ratio: LFn = (1 + (LF/HF) − 1) − 1, so individual LFn values contain no more information than individual LF/HF ratio values (Heathers, 2014). However, statistical results on them might differ due to the volatility of the LF/HF ratio when HF power approaches zero (Billman, 2013; Heathers, 2014).

Regarding time-domain variables, SDNN reflects all the cyclic components responsible for HRV (Laborde et al., 2017). Lastly, the root mean square of successive differences (RMSSD), the percentage of RR intervals which exceed 50 ms from the previous one (pNN50), and the SD of successive differences (SDSD) are correlated with the HF band, so vagal activity is considered to be in the physiological origin of these three variables. Of these, RMSSD is normally preferred since it is less influenced by respiration (Laborde et al., 2017). Despite the former caveats in their interpretation, the study of HRV indices is an area of great interest as they provide a low-cost and non-invasive window into ANS regulation of the heart.

### Data Acquisition and Processing

HRV analysis was performed on 3-min running windows taken every 30 s. In each window, outlier RR intervals were identified by imposing a limit on the derivative of the instantaneous HR, which cannot exceed a time-varying threshold based on the median of its previous values (Mateo and Laguna, 2003). Only those windows with less than 10 outliers (always below 5% in this study) were considered for further analysis. Two different HRV representations were used for time and frequency domain HRV indices estimation.

For time domain indices, the RR series was used, after correction of identified outlier RR values using the interpolation proposed in Mateo and Laguna (2003). The following indices were computed: MHR, RMSSD, pNN50, SDSD, and SDNN (Malik et al., 1996).

For frequency domain indices, the HRV representation used is the modulating signal, based on the heart timing signal, since it was shown to outperform other HRV representations for frequency domain indices estimation (Mateo and Laguna, 2000). The modulating signal, assumed to carry information from the ANS, was estimated from the beat occurrence time series, derived from the recorded RR intervals, based on the time-varying integral pulse frequency modulation model (Bailón et al., 2011). First, the instantaneous HR signal was estimated, sampled at a sampling frequency *F*s = 4 Hz, and denoted by *d*_HR_(*n*). Subsequently, the time-varying MHR signal, *d*_MHR_(*n*), was estimated by low-pass filtering *d*_HR_(*n*) with a cutoff frequency of 0.03 Hz. The modulating signal, *m*(*n*), was estimated by normalizing the HRV signal, *d*_HRV_(*n*) = *d*_HR_(*n*) − *d*_MHR_(*n*), by the time-varying MHR, i.e., *m*(*n*) = *d*_HRV_(*n*)/*d*_MHR_(*n*). Note that the modulating signal *m*(*n*) is adimensional. The purpose of this normalization is to alleviate the effect that changes in MHR have on HRV (Bailón et al., 2011). Then, the power spectral density (PSD) of the modulating signal *m*(*n*) was estimated using Welch periodogram with internal window of 2 min and 50% overlap. The power in the following bands was estimated: (i) LF, from 0.04 to 0.15 Hz; (ii) HF, from 0.15 to 0.40 Hz; (iii) extended HF (HFext), from 0.15 to half the MHR, to avoid misestimation of the HF component when respiratory rate is above 0.4 Hz (24 breaths per minute; Bailón et al., 2007). The LFn power was computed by dividing LF power by the sum of LF and HF powers (LFn), and the extended LFn was determined by dividing LF by the sum of LF and HFext (LFn_ext). Finally, the ratio between the LF and HF powers (LF/HF) and the ratio between LF and HFext powers (LF/HFext) were calculated.

As HRV analysis was performed on running 3-min windows taken every 30 s, the mean of each HRV variable in all running windows with less than 10 outlier RR values was computed to characterize each subject.

### Statistical Analysis

Descriptive values are presented as mean ± SD and HRV values are reported as median and (1st quartile–3rd quartile). The normality of data was checked with the Shapiro-Wilk test. Since the data distribution violated the assumption of normality required by parametric tests and could not be corrected by common transformations, a non-parametric analysis was used. To assess differences between the three age groups, Kruskal-Wallis test (non-parametric equivalent of one-way ANOVA) with Bonferroni correction was performed. The Dunn-Bonferroni *post hoc* method was used for pairwise comparisons. A multiple linear regression was performed to control for potential confounding effects like the body mass index (BMI). To evaluate the magnitude of the difference, effect size (ES) was calculated as: ES = *chi* ^2^/(k − 1), where *k* = total number of subjects. The difference was considered as small when ES < 0.2, small to medium when ES = 0.2–0.5, medium to large when ES = 0.5–0.8, and large when ES > 0.8 (Cohen, 1992).

A sub-analysis was carried out by following centenarians until their death and calculating Spearman’s correlation coefficient (*ρ*) between HRV variables and “time to death.” For the HRV variables showing significant correlation to “time to death,” centenarians were divided into two groups by setting a cut point that defined a high risk group containing one third of the centenarian population and a low risk group containing the remaining two thirds. The Mann-Whitney U test was used to compare differences between the statistical distributions of the two groups. Kaplan-Meier survival analysis was performed and the log-rank test was used to test survival differences between the two groups. Additionally, the value of HRV variables in predicting survival was determined by Cox proportional hazards analyses. Statistical analyses were performed using IBM SPSS (version 25; Chicago, IL, United States). The significance level was set at *p* < 0.05.

## Results

Table 1 shows the descriptive characteristics of the groups.

**Table 1:** Descriptive characteristics of the groups.

Variable	Young adults (*N* = 20)	Octogenarians (*N* = 18)	Centenarians (*N* = 17)
Age (years)	20.6 ± 2.3 (18–26)	84.1 ± 2.6 (80–88)	101.9 ± 1.9 (100–105)
Women (%)	100	100	76.5
BMI (kg/m^2^)	20.7 ± 1.9 (17–24)	27.0 ± 2.8 (22–31)	23.1 ± 3.4 (17–28)
Chronic diseases (%)^*^	Osteoarthritis	50	59
	CVD	44	53
	Dementia	11	47
	Diabetes	33	18
	AHT	61	53
	COPD	6	6
	Others	61	65
	Total number	3.3 ± 1.7 (0–5)	3.3 ± 1.0 (2–6)

* All young adults were healthy.

Scale values are mean ± standard deviation (SD) and min-max; BMI, body mass index; CVD, cardiovascular disease; AHT, arterial hypertension; COPD, chronic obstructive pulmonary disease.

**Figure 1:**
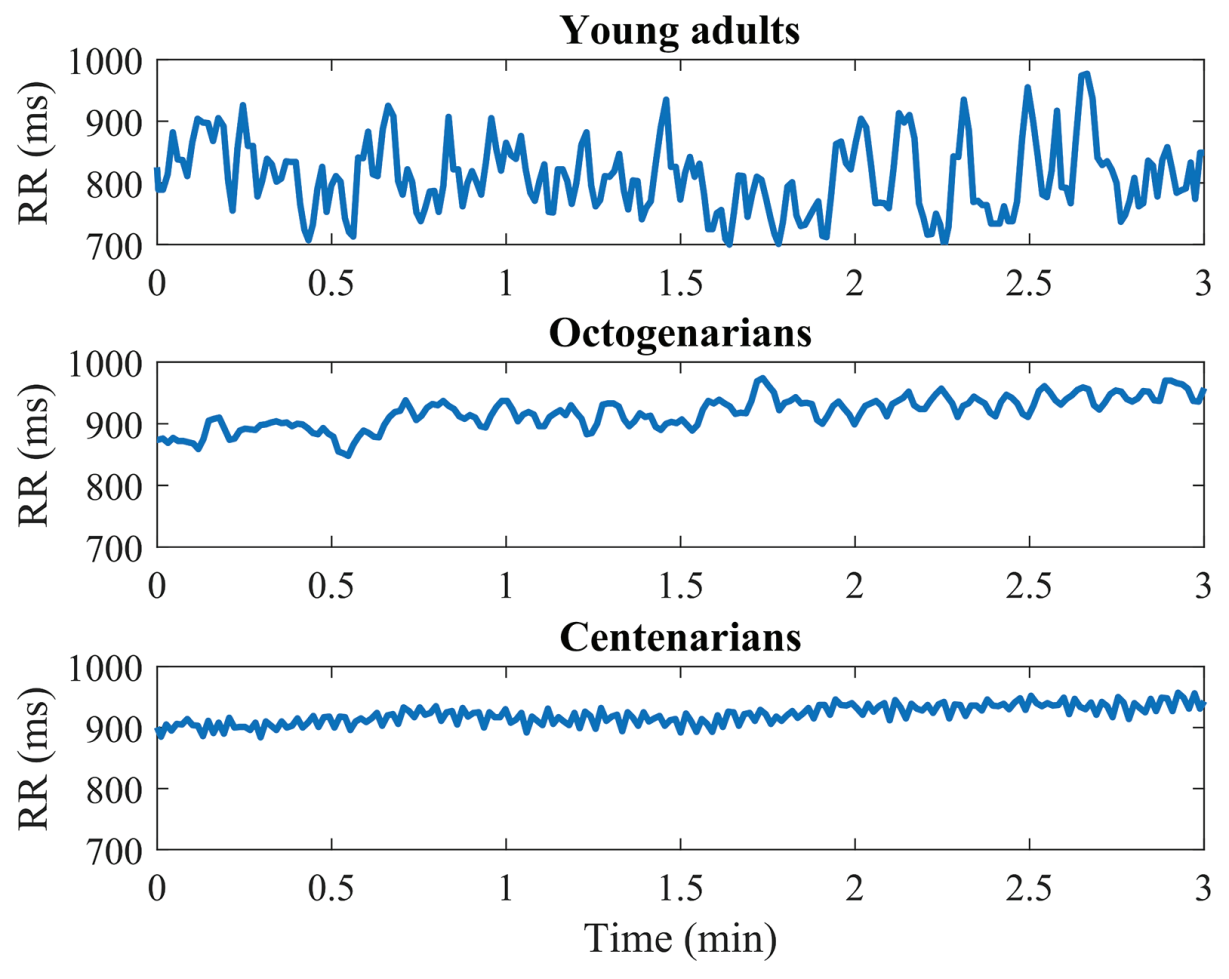
Example of the RR interval series for one subject from each age group.

An illustrative example of the RR interval series for each group is shown in Figure 1. Results obtained for the HRV variables in each age group are shown in Table 2. Differences between groups were only observed when analyzing the parasympathetic variables: RMSSD, pNN50, HF, HFext, and SDSD as well as LF and SDNN. All these variables decreased significantly with age (main effect: *p* < 0.05) but no statistically significant differences were found between octogenarians and centenarians.

**Table 2:** Differences between groups using Kruskal-Wallis non parametric test.

Variable	Young adults (*N* = 20)	Octogenarians (*N* = 18)	Centenarians (*N* = 17)	Main effect	
	Median (Q_1_–Q_3_)	Median (Q_1_–Q_3_)	Median (Q_1_–Q_3_)	*p*	ES
LF	0.0015 (0.0012–0.0031)^†^^‡^	0.0004 (0.0001–0.0017)	0.0002 (0.0001–0.0004)	<0.001^*^	0.441
LFn	0.5708 (0.5204–0.6266)	0.6131 (0.4608–0.7031)	0.5366 (0.3867–0.6456)	0.504	0.025
LFn_ext	0.5517 (0.4969–0.5997)	0.5701 (0.4286–0.6202)	0.4426 (0.2840–0.5887)	0.168	0.066
HF	0.0016 (0.0007–0.0032)^†^^‡^	0.0002 (0.0000–0.0009)	0.0001 (0.0001–0.0003)	<0.001^*^	0.414
HFext	0.0018 (0.0008–0.0035)^†^^‡^	0.0002 (0.0001–0.0010)	0.0002 (0.0001–0.0005)	<0.001^*^	0.367
RMSSD	57.97 (41.83–109.35)^†^^‡^	24.37 (10.14–67.29)	36.37 (18.71–50.55)	0.005^*^	0.194
pNN50	24.31 (12.61–41.24)^†^^‡^	2.62 (0.26–22.00)	6.88 (0.85–9.29)	<0.001^*^	0.283
SDSD	58.02 (41.91–109.59)^†^^‡^	24.41 (10.16–67.44)	36.44 (18.74–50.57)	0.005^*^	0.194
SDNN	68.13 (52.55–130.75)^†^^‡^	41.38 (17.41–82.03)	36.77 (21.25–45.90)	0.001^*^	0.276
LF/HF	1.44 (1.12–1.91)	1.78 (0.91–2.39)	1.36 (0.68–2.19)	0.650	0.016
LF/HFext	1.32 (1.03–1.61)	1.41 (0.80–1.75)	0.85 (0.43–1.60)	0.303	0.044
MHR	72.49 (65.87–79.05)	70.50 (64.44–76.62)	72.61 (62.23–85.50)	0.687	0.014

* *p* < 0.05.

† Different to octogenarian.

‡ Different to centenarian.

Values are expressed as median and (1st quartile–3rd quartile). LF, low frequency; LFn, normalized LF; LFn_ext, extended LFn; HF, high frequency; HFext, extended HF; RMSSD, root mean square of successive differences; pNN50, percentage of RR intervals which exceed 50 ms from the previous one; SDSD, SD of successive differences; SDNN, SD of the RR series; LF/HF, ratio between LF and HF; LF/HFext, ratio between LF and HFext; MHR, mean heart rate; *p*, *p*-value for Kruskal-Wallis test; ES, effect size.

We were able to follow up 14 centenarians until death. The three subjects with incomplete data were females but no significant differences with the 14 subjects included in the sub-analysis were found either in the descriptive variables or in the HRV variables. The only HRV variable that presented significant correlation with survival prognosis in centenarians (Table 3) was SDNN (*ρ* = 0.536, *p* = 0.048).

**Table 3:** Survival prognosis in centenarians (*N* = 14).

	LF	LFn	LFn_ext	HF	HFext	RMSSD	pNN50	SDSD	SDNN	LF/HF	LF/HFext	MHR
*ρ*	0.423	0.172	0.295	0.190	0.232	0.304	0.247	0.304	0.536	0.214	0.251	−0.082
*p*	0.131	0.557	0.305	0.516	0.426	0.290	0.395	0.290	0.048^*^	0.463	0.386	0.782

* *p* < 0.05.

LF, low frequency; LFn, normalized LF; LFn_ext, extended LFn; HF, high frequency; HFext, extended HF; RMSSD, root mean square of successive differences; pNN50, percentage of RR intervals which exceed 50 ms from the previous one; SDSD, SD of successive differences; SDNN, SD of the RR series; LF/HF, ratio between LF and HF; LF/HFext, ratio between LF and HFext; MHR, mean heart rate; *ρ*, Spearman’s correlation coefficient with “time to death”; *p*, *p*-value for Pearson’s correlation coefficient.

**Figure 2:**
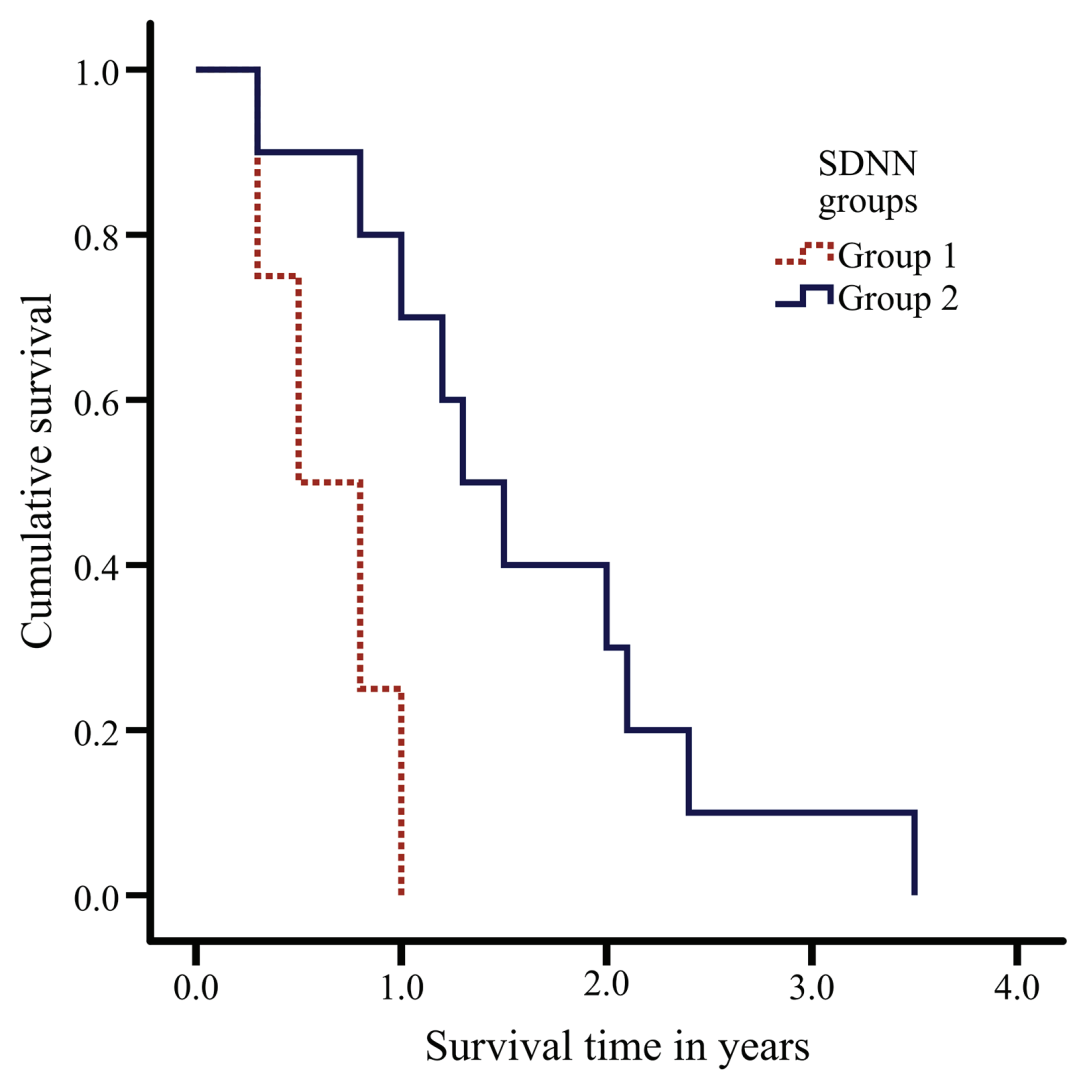
Kaplan-Meier curves depicting association between survival in centenarians (*N* = 14) and SDNN groups based on a cut point of 19 ms.

The 14 centenarians were divided according to the SDNN variable into two groups based on a cut point of 19 ms: Group 1 presenting low SDNN values (*N* = 4; 15 ± 4 ms, range: 10–18) and Group 2 presenting high SDNN values (*N* = 10; 49 ± 25 ms, range: 27–110). The difference between the statistical distributions of the two groups was statistically significant. Group 2 was associated with greater survival (1.6 ± 0.9 years, range: 0.3–3.5) than Group 1 (0.6 ± 0.3 years, range: 0.3–1.0) in Kaplan-Meier analysis (*Log-rank test* = 0.010, Figure 2). Mortality risk in Group 1 was five times higher (*p* = 0.028; Hazard Ratio = 5.72) than in Group 2. No relation was found between subjects’ age at RR recording time and time to death (*p* = 0.477).

## Discussion

The present study shows that parasympathetic time-domain HRV measures as well as SDNN and LF all decrease with age; moreover, other variables such as LFn or LF/HF ratio do not indicate differences between age groups. In relation to survival prognosis, SDNN was the only HRV measure showing moderate correlation (*ρ* = 0.5–0.7) with time to death in centenarians, with SDNN values below 19 ms being associated with early mortality (≤1 year) in centenarians (Hazard Ratio = 5.72).

### HRV Measures

When analyzing the main parasympathetic HRV variables (RMSSD, pNN50, HF, and SDSD; Malik et al., 1996; Laborde et al., 2017), a clear decrease with age is observed in all of them, with very remarkable differences between young adults and older adults but without significant differences between octogenarians and centenarians. This age-related decrease can be appreciated in the illustrative examples of Figure 1 and has already been reported by other authors (Umetani et al., 1998; Bonnemeier et al., 2003; Abhishekh et al., 2013). Also, to the best of our knowledge, this is the first time that it has been described in centenarians. It should be emphasized that our results differ from those of Paolisso et al., Zulfiqar et al., and Almeida-Santos et al. since they establish a parasympathetic nadir at 75–80, 70–79, and 60–69 years, respectively, and our study indicates that parasympathetic HRV variables continue to decrease in centenarians (Piccirillo et al., 1998; Zulfiqar et al., 2010; Almeida-Santos et al., 2016). There are several possible explanations for our sample of centenarians not showing a reversal of the decrease in parasympathetic function. First, erratic rhythms may have a confounding effect on age-related changes in parasympathetic HRV indices (Nicolini et al., 2012), which is why our study only analyzes RR segments free of erratic patterns, allowing the presence of no more than 10 outlier values in each 3-min window of analysis. Second, although the BMI of the octogenarian sample was significantly higher, BMI had no effect as a confounding variable (*p* > 0.05 in the multiple linear regression). Some studies have found reduced HRV in underweight and overweight adult women (Triggiani et al., 2017; Gerardo et al., 2019), but studies in the literature investigating the oldest old are scarce. The lower BMI in the centenarian sample could be an indicator of healthy body composition but also a simple consequence of age-associated sarcopenia or osteoporosis. On the other hand, previous studies in centenarians have been very restrictive in the selection of subjects, including only very healthy and independent subjects (Piccirillo et al., 1998; Paolisso et al., 1999; Zulfiqar et al., 2010), which may involve a selection bias (Tan et al., 2019). The parasympathetic decrease in centenarians found in our study could, thus, be more representative of a natural exhaustion of allostatic systems related to age.

As already mentioned, the interpretation of the standard HF band (0.15–0.4 Hz) is compromised when respiratory rate does not fall within this band (9–24 bpm). Since breathing was not monitored, power in the extended HF band (0.15-half MHR) was computed to account for respiratory rates that might exceed 24 bpm, as suggested in Bailón et al., 2007. As it can be seen in Table 2, results of the standard HF band were parallel to those of the extended HF band, suggesting that in this database, respiratory rates were in the standard HF band (0.15–0.40 Hz). Therefore, HF could be considered as a measure of the vagal tone (Laborde et al., 2017).

SDNN can be considered as an indicator of global autonomic regulation, although it has been claimed that in short-term recordings, the primary source of its variations is parasympathetically-mediated RSA (Shaffer and Ginsberg, 2017). In agreement with previous studies (Zulfiqar et al., 2010; Almeida-Santos et al., 2016; Sammito and Böckelmann, 2016), SDNN values decline with age, further reflecting an age-dependent decline in ANS activity. LF results were in the same line as the parasympathetic HRV variables and SDNN, probably because the recording was made while sitting upright during resting and under these conditions the primary contributors to HRV have been suggested to be related to parasympathetic and baroreflex activity rather than to sympathetic activity (Shaffer and Ginsberg, 2017).

Other HRV variables, LFn and LF/HF, whose physiological interpretation is usually controversial, have been additionally investigated in our study, neither of them showing statistically significant differences between groups.

### HRV and Survival Prognosis in Centenarians

In recent decades, HRV has been confirmed as a strong, independent predictor of morbidity and all-cause mortality (Billman, 2011; Kemp et al., 2017). To investigate HRV variables that may be associated with survival prognosis in centenarians, we followed up subjects until death. Only SDNN showed significant correlation (*ρ* = 0.536, *p* = 0.048) with survival prognosis in centenarians. The group of centenarians with low SDNN values presented five times greater mortality risk than centenarians with high SDNN values. In the framework of the research topic “Horizon 2030: Innovative Applications of Heart Rate Variability,” we discuss about HRV and exceptional longevity. However these results should be read with perspective, as the sample of centenarians followed until death is heterogeneous in gender, including 4 men and 10 women.

Since Kleiger et al. set the basis for the use of HRV in post-acute myocardial infarction risk stratification in 1987, SDNN is considered as a “gold standard” when recorded over a 24-h period. SDNN values below 50 ms are classified as unhealthy, 50–100 ms as compromised health, and above 100 ms as healthy (Kleiger et al., 1987). According to Bilchick et al. (2002), each 10-ms increase in SDNN confers a 20% decrease in risk of mortality. SDNN is the only variable presenting significant correlation with time to death in our cohort of centenarians. In particular, SDNN <19 ms turns out to be indicative of early mortality (≤1 year). Of note, one subject presented a value of SDNN of 110 ms and was the one who lived the longest time (3.5 years) calculated from the time point when RR was recorded.

It should be noted that there are other RR-derived variables that have been related to increased mortality risk in the literature. The fact that they have not been found to be associated with time to death in our study could be due to the small sample of our cohort or to the particular characteristics of the studied centenarians. A classic example is high resting HR (Zhang et al., 2015). Additionally, a recent meta-analysis has established LF/HF ratio and SDNN as two of the variables with greater potential as predictors of mortality (Sen and McGill, 2018). Shimizu et al. (2002) have also observed the relevance of LF/HF ratio in a cohort of 27 centenarians. Finally, LF is one of the most controversial HRV indices in the literature. Some studies, such as the Framingham Heart Study, have associated a 1-SD decrement in LF with 1.70 times greater hazard for all-cause mortality (Tsuji et al., 1994). On the other hand, cross-sectional studies in healthy centenarians have reported that high LF values are associated with increased mortality risk (Piccirillo et al., 1998; Paolisso et al., 1999).

### Centenarians and the “Neurovisceral Integration Across a Continuum of Time” Framework

Centenarians are considered to be a model of healthy and successful aging. It is well known that exceptional longevity is a partially inheritable phenotype that could be explained in 20–35% by the genetic load (Rea et al., 2016). Consequently, it could be that the ANS of the centenarians had a greater and innate adaptation level, and therefore they will take 20 years more than the general population to reach a level of depletion of the allostatic systems related to mortality. On the other hand, another feasible explanation would be that centenarians have healthy behaviors that allow them to experience a less marked decrease in the function of the ANS. Non-genetic factors, including diet, physical activity, health habits, and psychosocial factors contribute approximately 50% of the variability in human lifespan (Rea et al., 2016).

Recently, Kemp et al. (2017) published a theoretical framework called “Neurovisceral Integration Across a Continuum of Time (NIACT)” where they propose that the function of the vagus nerve, indexed by resting-state HRV, plays a regulatory role on a variety of allostatic systems, therefore contributing to an increase or decrease in the risk of future morbidity and mortality. NIACT proposes that while age decreases vagal function, there are many interventions that may be applied to contend such decreases including health behavior, meditation, and positive psychological interventions (Kemp et al., 2017). Health behaviors related to improvements in HRV are similar to those that characterize the lifestyle of centenarians in different populations: regular physical activity, dietary habits, no drinking, and no smoking (Ozaki et al., 2007; Kim et al., 2012; Wu et al., 2017). But psychological moments are also a key element in the NIACT framework, and in the same way, active engagement in community activities, high levels of self-perceived well-being, and satisfaction with life are defining elements of the centenarian population (Ozaki et al., 2007; Kim et al., 2012; Wu et al., 2017; Hitchcott et al., 2018; Yorgason et al., 2018). Therefore, the characteristic lifestyle of centenarians would imply a greater resilience, indexed by greater variability of the HR and, as described above, higher SDNN values would mean better survival prognosis in centenarians.

### Strengths and Limitations

The main strength of the present study is the exceptionality of the sample, considering than being centenarian is a rare phenotype (17.3 centenarians per 100.000 inhabitants; Teixeira et al., 2017). Secondly, centenarians were followed up to death and our study proposes SDNN <19 ms as a cutoff point to define a marker of early mortality (≤1 year), which is obtained from short-term measurements, thus more suitable for ambulatory care and patient monitoring. Given its ease of recording, short-term variability allow measurements under homogeneous conditions, enabling the control of confounding factors and the reproducibility of the study (Li et al., 2019). Moreover, RR measurements were acquired using a validated device and processed with methods that allow better identification of the erratic patterns.

On the other hand, one of the main limitations was gender heterogeneity in the centenarian group, with 76.5% of our centenarians being women. A meta-analysis has highlighted that women show greater vagal activity compared to men, noting the following possible etiological factors: estrogen, oxytocin, and neural control (Koenig and Thayer, 2016). Our sample, however, is very similar to the overall centenarian population in Europe (83.5% women; Teixeira et al., 2017), and sex differences have been reported to disappear in the last age decades, especially in short-term HRV, presumably by the hormonal restructuring especially caused by the menopause in women (Bonnemeier et al., 2003; Voss et al., 2015; Koenig and Thayer, 2016). Indeed, when the statistical analysis of our study was performed by excluding men (*N* = 4) from the sample of centenarians, results were similar to those reported in Table 2. Finally, recording conditions should be taken into account before generalizing the results. For example, paced breathing was not considered in our work. Under resting conditions, the respiratory rate (9–24 bpm) is expected to be in the 0.15–0.40 Hz band (Shaffer and Ginsberg, 2017), but processing should account for the possibility that the respiratory rate goes outside this frequency band, as performed in the present work. A within-subject repeated measure design would have contributed to assess the reproducibility of our evaluations. In future research, more representative samples of centenarians would allow to confirm the results obtained by this study.

In conclusion, HRV indices reflecting parasympathetic outflow (RMSSD, pNN50, HF, and SDSD) as well as SDNN and LF all present an age-related reduction, which could be representative of a natural exhaustion of allostatic systems related to age. Moreover, low SDNN values (<19 ms) are indicative of early mortality (≤1 year) in centenarians. HRV seems to play a role in exceptional longevity, which could be accounted for by centenarians’ exposome.

## Data Availability Statement

The raw data supporting the conclusions of this article will be made available by the authors, without undue reservation.

## Ethics Statement

The studies involving human participants were reviewed and approved by Clinical Research Ethics Committee of the University Hospital of Alcorcón (ID of the approval: 16/50). The patients/participants provided their written informed consent to participate in this study.

## Author Contributions

NG designed the overall study. AS-L, GR-R, and AH-V collected the data. All authors contributed equally in the interpretation and analysis of the data, revision of manuscript for important intellectual content and have read and approved the final version.

### Conflict of Interest

The authors declare that the research was conducted in the absence of any commercial or financial relationships that could be construed as a potential conflict of interest.
